# A Highly Tailored Text and Voice Messaging Intervention to Improve Medication Adherence in Patients With Either or Both Hypertension and Type 2 Diabetes in a UK Primary Care Setting: Feasibility Randomized Controlled Trial of Clinical Effectiveness

**DOI:** 10.2196/16629

**Published:** 2020-05-19

**Authors:** Aikaterini Kassavou, Venus Mirzaei, James Brimicombe, Simon Edwards, Efthalia Massou, A Toby Prevost, Simon Griffin, Stephen Sutton

**Affiliations:** 1 The University of Cambridge Cambridge United Kingdom; 2 Imperial College London Cambridge United Kingdom

**Keywords:** medication adherence, hypertension, type 2 diabetes, text messaging, interactive voice response

## Abstract

**Background:**

The efficacy of a highly tailored digital intervention to support medication adherence and feasibility to support clinical effectiveness as an adjunct to the primary care setting has not been evaluated.

**Objective:**

This trial aimed to assess the behavioral efficacy of a highly tailored digital intervention to support medication adherence and to evaluate the feasibility of its clinical effectiveness, in patients with either or both hypertension and type 2 diabetes. We also examined quality of life and mechanisms of behavior change. Intervention fidelity, engagement, and satisfaction were also explored.

**Methods:**

This was a multicenter, individually randomized controlled trial of 2 parallel groups: an intervention group that received a highly tailored text message and interactive voice response intervention for 12 weeks, and a control group that received usual care. Medication adherence was measured using self-reports and assessor-blinded practice records of a repeat prescription. Systolic blood pressure and glucose levels were assessed by nurses blinded to group allocation during practice visits at 3 months follow-up. Questionnaires obtained data to assess intervention mechanisms of action and satisfaction and digital log files captured data to evaluate fidelity and engagement.

**Results:**

A total of 135 nonadherent patients (62/135, 46% female; 122/135, 90.3%; aged above 50 years) were randomly allocated in the intervention (n=79) or in the control group (n=56); of whom 13% (18/135) were lost at follow-up. Medication adherence was significantly improved in the intervention group compared with the control group (*t*_116_=2.27; *P*=.02, 2-tailed). Systolic blood pressure was 0.6 mmHg (95% CI −7.423 to 6.301), and hemoglobin A_1c_ was 4.5 mmol/mol (95% CI −13.099 to 4.710) lower in the intervention group compared with the control group. Changes in intentional nonadherence and nonintentional nonadherence explained the improvements in medication adherence in the intervention group (beta=.074, SE=0.464; *P*=.04), but not in the control group (beta=.00, SE 1.35; *P*=.37). The intervention had 100% fidelity, a median of 12 days of engagement, and 76% overall satisfaction.

**Conclusions:**

Our trial is the first that has been conducted in the United Kingdom and showed that among nonadherent patients with either or both hypertension and type 2 diabetes, a highly tailored digital intervention was effective at improving treatment adherence and feasible to obtain clinically meaningful outcomes. Changes in intentional and nonintentional nonadherence predicted the improvements in medication adherence. The intervention had high fidelity, engagement, and satisfaction. Future research using a rigorous design is needed to evaluate the clinical effectiveness and cost-effectiveness of the intervention in primary care.

**Trial Registration:**

International Standard Randomized Controlled Trial Number (ISRCTN) 10668149; http://www.controlled-trials.com/ISRCTN10668149.

## Introduction

### Background

The clinical management of cardio-metabolic conditions, like hypertension and type 2 diabetes mellitus, is one of the most common consultations in primary care [1,2] and pharmacotherapy is an essential part of effective management of these conditions [3]. However, many patients do not adhere to their prescribed medication regimen [4], leading to reduced treatment efficacy, increased risk of complications, additional consultations, and hospital admissions [5,6]. Medication adherence can significantly reduce these risks and prevent health complications [7,8]. It could also result in health care savings, with estimates from the United Kingdom of approximately US $115.4 million preventable cost being spent annually, due to only nonadherence to antihypertensive medications [9].

### Setting

Primary health care providers can facilitate treatment adherence, but their time is limited and expensive. Given the growing prevalence of hypertension, the associated comorbidities, and the aging population, it is likely that there will be an increased need for health care resources to support medication adherence. Digital interventions are promising strategies to support adherence [10,11] and can be highly tailored and acceptable adjunct to primary care consultations [12]. Telephone-based interventions that deliver text and voice messages may have a wider reach as they can be delivered to any device, even in places of low network coverage, and are accessible to people from all socioeconomic backgrounds and age groups. We have therefore developed the Medication Adherence for Patient’s Support intervention, a behavioral intervention that uses text and voice messages, to support medication adherence in patients with either or both hypertension and type 2 diabetes as an adjunct to primary care consultations.

### Aims and Objectives

This study aimed to assess if patients with either or both hypertension and type 2 diabetes, who used the intervention for 3 months in addition to usual care, had improved medication adherence and if they differed in terms of systolic blood pressure or hemoglobin A_1c_, and quality of life at 3 months follow-up. We have also assessed intervention mechanisms of action, as well as intervention fidelity, engagement and satisfaction. However, this feasibility trial primarily aimed to attain evidence about the feasibility of obtaining medication-related clinical outcomes to inform a larger effectiveness and cost-effectiveness trial in primary care.

## Methods

### Study Design

We used an individually randomized controlled trial of unequal 3:2 allocation ratio with a 3-month follow-up. We used unequal allocation ratio to increase the information obtained about the intervention. Intervention group patients received highly tailored and interactive text and voice recognition messages for 12 weeks, as an adjunct to usual care. The control group received usual care only [13].

### Study Setting

Participants were recruited over 7 months, from March to September 2018, from 8 primary care practices in the East of England. We selected a wide range of primary care practices from different areas of deprivation (based on Index of Multiple Deprivation), and at least 50% in highly deprived areas, to obtain information about the reach and implementation of the intervention in different sites, and to increase the scalability of the intervention to a larger trial. Follow-up data collection was conducted between May 2018 and February 2019.

### Participant Recruitment and Eligibility Criteria

A member of the practice staff screened medical databases against the inclusion criteria to identify eligible patients. Patients were eligible when they met all the following criteria: (1) were 18 years or older, (2) had a diagnosis of hypertension, type 2 diabetes mellitus, or both health conditions; (3) had been prescribed at least 1 antihypertensive medication or glucose lowering medication as documented in practice records for a period of 3 to 6 months before recruitment; and (4) had either or both poorly controlled blood pressure (controlled by age group) and glucose levels as logged in their medical records, or had gaps in collecting repeat prescriptions during the 6 months before the study invitation. When possible, patients’ digital literacy was screened for inclusion.

A general practitioner screened the generated list of potentially eligible patients against the exclusion criteria to confirm eligibility. Patients were excluded when they met any of the following criteria: (1) were taking part in another medication adherence or digital intervention or (2) had a health condition that could impair their participation in the study.

Eligible patients were invited to the study by the primary care practices using text messages, postal invitations, and follow-up calls to postage invitations, or were recruited opportunistically during usual care consultations (eg, blood pressure checks and medication reviews) by health care providers.

### Study Procedures

Interested patients were invited and attended baseline consultations with a health care provider (a practice nurse or a health care facilitator), where they were screened for eligibility (eg, digital literacy) and asked to provide informed consent. Baseline consultations involved the completion of a baseline questionnaire, measuring medication adherence, quality of life, and theoretical determinants associated with intentional nonadherence and nonintentional nonadherence (eg, necessity beliefs about medication taking and self-efficacy) and were facilitated by the health care provider. Responses to the baseline questionnaire were included in a webpage (which consisted of the tailoring algorithm, the intervention schedule, the intervention messages, and the inbound calls) to further inform the tailored intervention to those allocated to receive the digital intervention.

After the end of the consultations, patients were randomized to intervention or control groups and were sent a text message or a letter with information about which group they are allocated in. Further, intervention group patients completed information (on a webpage or verbally during phone calls; depending on their digital literacy) about their preferred times and frequency to receive the intervention messages. They were also provided with more information on how to use the digital delivery modes. All patients were provided with additional information about the trial procedures. Baseline consultation was conducted few days before each patient’s repeat prescription was due for collection.

### Intervention

Intervention group patients received highly tailored and interactive text and voice recognition messages for 12 weeks, initiated the day their repeat prescription was due for collection. The intervention development was guided by the theoretical framework that distinguishes between intentional and nonintentional nonadherence [14], previous evidence [11,12,15], and included behavior change techniques and strategies mapped onto either or both intentional and nonintentional nonadherence. Nonintentional nonadherence refers to patients not taking their medications as prescribed because they forget or misunderstand the recommendations. Intentional nonadherence refers to patients not taking their medications as prescribed because they decide to take less medication or miss a dose or a day of their medications. The intervention aimed to improve medication adherence by employing behavior change strategies to modify either or both intentional and nonintentional nonadherence.

The interactive element of the intervention was utilized for dynamic tailoring: patients could record a personalized implementation intention plan to further tailor intervention content and be grouped to receive more habit formation or self-monitoring advice based on records and responses obtained during the intervention. We have also adopted a flexible approach to intervention delivery, providing participants with functions to change delivery options (eg, decrease or increase the frequency of messages or stop the messages). Details of the intervention development are published elsewhere [12].

### Outcomes

This trial aimed to assess the behavioral efficacy of a highly tailored digital intervention to support medication adherence in patients with either or both hypertension and type 2 diabetes, and its feasibility to support medication-related clinical outcomes. We also examined mechanisms of action. Recruitment and attrition rate and intervention fidelity, engagement, and satisfaction were also reported.

Primary outcomes were the efficacy of the intervention to support medication adherence and the feasibility of its clinical effectiveness at a 3-month follow-up (T2). Secondary outcomes included the mechanisms by which the intervention supported medication adherence, as well as intervention engagement and satisfaction.

Efficacy was measured using 2 self-reported items of adherence (ie, days of adherence during the past week and percentage of adherence during the past month) at T2, and repeat prescription claims during the last 2 intervention months before T2. A subsample of patients (n=9 intervention and n=2 control group) were randomly selected and invited to use a Medication Event Monitoring System caps for a duration of 7 consecutive days after T2. Medication Event Monitoring System is an electronic pill-bottle cap that registers the date and time of bottle opening [16]. Patients were provided with information about how to use the Medication Event Monitoring System and the number and specific medications to include in the container during follow-up consultations (see Multimedia Appendix 1). Practice records raw data (ie, dates of prescription claims) were extracted by a member of the practice staff from medical records and Medication Event Monitoring System data were extracted by a member of the research team; both nursing and research team staff were blinded to group allocation.

Clinical outcomes were measured at T2 by nurses blinded to group allocation during practice visits. Blood pressure was measured in patients prescribed antihypertensive medications and hemoglobin A_1c_ from those prescribed glucose-lowering medications. Blood pressure was measured 3 times with 1-minute intervals using calibrated blood pressure digital monitor devices (eg, Omron). The last 2 readings were included in the analysis. Blood samples were anonymized and sent to the Addenbrookes Hospital Pathology laboratory for analysis of hemoglobin A_1c_.

Quality of life was measured using the 5-level EQ-5D [17]. Medication Adherence Rating Scale [18] was used to measure intentional nonadherence (4-items) and nonintentional nonadherence (1-item). Feasibility was assessed by the recruitment and attrition rate (percentage of people who responded to the invitation and those randomized and the percentage of people who attended consultations at follow up).

Intervention fidelity was measured by calculating the proportion of messages received out of those messages scheduled per patient, for the duration of the 12 weeks intervention. Intervention engagement was calculated using the median number of days patients interacted with the intervention during the 12-week intervention period. Data were captured objectively by digital log files and extracted by a member of the research team.

### Sample Size

The sample was selected to obtain evidence about the feasibility to implement a cost-effectiveness trial in primary care. It aimed to provide evidence about the potential effectiveness of the intervention, and the values needed to estimate clinically meaningful outcomes (eg, mean and CIs for systolic blood pressure and hemoglobin A_1c_). We included more participants in the intervention group, to obtain more information about the mechanisms by which the intervention supported medication adherence, and about the intervention engagement and satisfaction.

### Randomization and Blinding

Block randomization (4 blocks, each of size of 25) was conducted to ensure random group allocation. The random sequence was generated by a centralized Web-based service [19] and was stratified in 2 important confounders: treatment adherence, as measured by the Medication Adherence Rating Scale, and burden of pills. Medication Adherence Rating Scale threshold of 24 was selected to indicate low (<24) or high (≥24) adherence. A burden of pills ratio of 10:6 (10 tablets: 6 different health conditions) was selected to indicate low (<10:6) or high (≥10:6) burden of pills; and the ratio was based on our pilot studies [12].

Data to calculate the burden of pills ratio were extracted from medical records, and Medication Adherence Rating Scale was self-reported. Both measures were entered into the randomization webpage by a member of the research team, after the completion of baseline consultations.

Health care providers were blinded to group allocation during baseline consultations. Participants were partly blinded during the baseline consultations as the purpose of the study was explained to them (trial videos in Multimedia Appendix 1).

### Data Analysis

The analysis was performed based on complete cases at follow-up, excluding missing data. That is, only participants who completed the outcome measures at follow-up were included in the analysis. Histograms were used to explore continuous variables’ distribution, and the Levene test was used to assess the assumption of equality of variance between groups. A *t*-test was used to investigate the differences between independent groups, and when its assumptions were not met, a nonparametric Mann-Whitney test for independent samples was conducted. Descriptive statistics (eg, means, SD, and percentages) was used for comparisons between groups, and for describing fidelity, engagement, and satisfaction with the intervention. A subgroup analysis was performed to explore differences in outcomes due to missing data: we compared if there were statistically significant differences between responders and nonresponders at each of the baseline and follow-up outcome measures. Subgroup analyses were performed on the total sample size. Multivariable regression analysis was performed to explore the mechanisms by which the intervention supported behavior change and control of medication-related clinical outcomes. The analysis was conducted in December 2019 using STATA.

### Ethical Consideration

The study protocol was approved by the Ethics Committee of East of England, Essex Research Ethics Committee (REC Reference number 17/EE/0203) and Health Research Authority. Written informed consent was obtained from all study participants.

## Results

### Recruitment

A total of 4468 eligible patients were invited to the study. From those, 256 expressed interest to participate (a 5.7% response to the invitation), 140 were invited and attended consultations, and 135 were deemed eligible and randomized to the intervention (n=79) or control (n=56) group.

### Randomization

The groups were very similar on baseline variables used for minimization and for most other variables. The majority (122/135, 90.3%) of patients were above 50 years of age (Table 1). Intervention group patients self-reported taking more pills per day compared with usual care group patients. This variable was not accounted for minimization, because an objective measure of burden of pills was selected (eg, burden of pills ratio extracted from medical records) instead of self-reports.

**Table 1:** Patients characteristics at baseline.

Variables^a^		Intervention group (n=79)	Usual care group (n=56)	*P* value	
**Index of multiple deprivation, n (%)**					**N/A^b^**
	20-30(most deprived)	9 (11.4)	6 (10.7)		
	30-40	46 (58.3)	31 (55.3)		
	40-50	0 (0)	0 (0)		
	50-60	6 (7.6)	4 (7.2)		
	60-70	6 (7.6)	8 (14.3)		
	70-80 (least deprived)	12 (15.1)	7 (12.5)		
**Age (years), n (%)**					**.37**
	18-29	0 (0)	1 (1.8)		
	30-39	4 (5.1)	0 (0)		
	40-49	5 (6.3)	3 (5.4)		
	50-59	15 (19)	13 (23.2)		
	60-69	27 (34.2)	15 (26.8)		
	70-79	26 (32.9)	20 (35.7)		
	80+	2 (2.5)	4 (7.1)		
**Gender, n (%)**					**.90**
	Male	40 (50.7)	33 (59)		
	Female	39 (49.3)	23 (41)		
**Employment, n (%)**					**.47**
	Full-time	22 (27.8)	10 (17.8)		
	Part-time	12 (15.2)	3 (5.4)		
	Unemployed	1 (1.2)	1 (1.8)		
	Unable to work due to disease	3 (3.8)	7 (12.5)		
	Retired	41 (52)	35 (62.5)		
Number of pills prescribed to take per day, self-reported, mean (SD)		8.13 (6.21)	5.95 (5.14)	.03	
Number of different pills prescribed to take per day, self-reported, mean (SD)		5.74 (3.67)	4.57 (3.52)	.06	
**Health condition, n (%)**					**N/A**
	Hypertension	42 (53.2)	38 (67.9)		
	Type 2 diabetes	32 (40.5)	16 (28.6)		
	Comorbidities of hypertension, type 2 diabetes, and cholesterol	5 (6.3)	2 (3.5)		
**Medication adherence, mean (SD)**					
	Number of days of adherence, last week	6.46 (1.23)	6.38 (1.29)	.73	
	Percentage of adherence, last month	91.19 (13.11)	93.98 (33.32)	.49	
	Medication Adherence Rating Scale	22.78 (2.81)	23.25 (2.32)	.30	
	Repeat prescription	0.97 (0.20)	0.95 (0.26)	.64	
**Quality of life, mean (SD)**					
	EQ-5D^c^, 5-items	1.71 (0.76)	1.74 (0.75)	.86	
	EQ-5D, total health	76.01 (19.68)	47.83 (21.56)	.74	

^a^Data are reported as means (SD) or number (percentage). Health condition: main comorbidities were extracted from prescription data. Index of Multiple Deprivation was calculated based on general practice postcode; 10: low Index of Multiple Deprivation to 100: high Index of Multiple Deprivation. Repeat prescription was defined and calculated: supply of medication claimed by the patients, excluding the next prescription day the medication was claimed, and divided by the number of days of assessment period. Assessment period at baseline refers to the supply claimed by each patient before the start of the study (ie, consent process). Assessment period at follow-up refers to the last 2 months of the study. Ratio was calculated per patient due to different denominators (eg, supply prescribed to be issued every 28 or 56 days). The overall adherence value was calculated by averaging each patient’s ratio and dividing by the total number of patients.

^b^N/A: not applicable.

^c^EQ-5D-5L: descriptive system of health-related quality of life states consisting of five dimension (mobility, self-care, usual activities, pain/discomfort, anxiety/depression) each of which can take one of five responses. The response record five levels of severity (no problems/slight problems/moderate problems/ severe problems/ extreme problems) within a particular EQ-5D dimension.

### Attrition Rate

**Figure 1:**
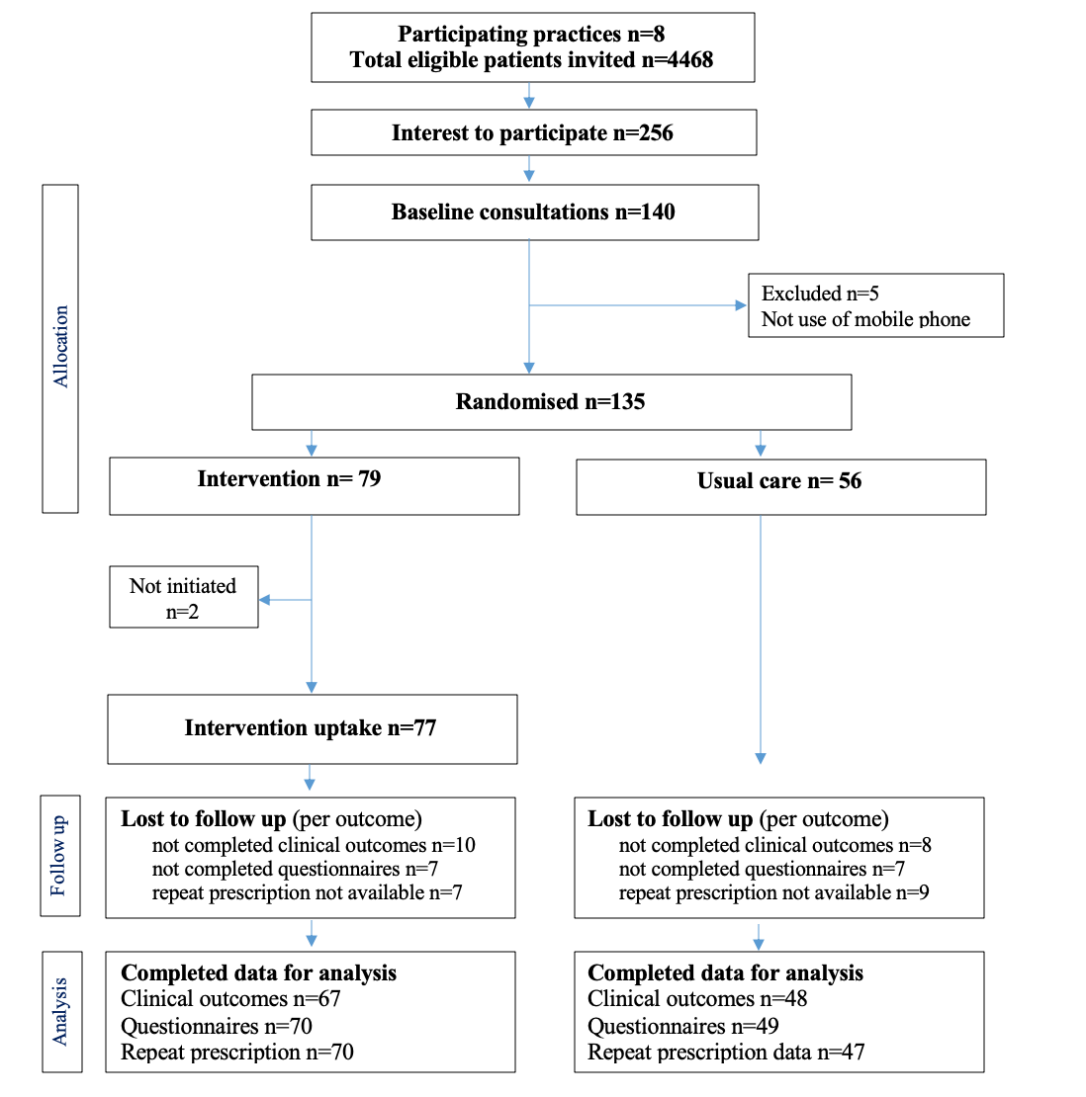
Trial CONSORT patients flow diagram.

At 3 months follow-up, 88% (119/135) of participants completed the self-reported questionnaires and 85% (115/135) completed practice visits and provided clinical outcomes. Repeat prescription data were obtained for 87% (117/135) of the recruited patients (Figure 1).

### Intervention Retention Rate

Of those randomized to the intervention group, 97% (77/79) initiated the intervention: intervention uptake was defined by the number of participants who responded to the intervention messages during the first week of the intervention. Reported reasons for not initiating the intervention after group allocation was hospitalization. Intervention drop out was 2.5%, defined as the number of participants requesting to stop the intervention; and all drop out was captured during the first 4 weeks of the intervention.

### Outcomes

The analysis found statistically significant difference in the repeat prescription adherence (*t*_116_=2.27; *P*=.02; mean 0.99 [SD 0.11] for the intervention group vs 0.92 [SD 0.21] for the control group), days of adherence (*t*_112_=2.37; *P*=.02; mean 6.85 [SD 0.47] vs 6.36 [SD 1.59]), Medication Event Monitoring System (*t*_10_=4.04; *P*<.001; mean 6.05 [SD 2.29] vs 3.5 [SD 4.94]), and percentage of adherence (*t*_112_=1.69; *P*=.05; mean 96.64 [SD 5.60] vs 91.89 [SD 18.60]) between groups, suggesting improvements in the intervention group. Nonstatistically significant differences in Medication Adherence Rating Scale (*t*_104_=−0.24; *P*=.98; mean 23.66 [SD 1.99] vs 23.67 [SD 2.33]) between groups were found (Table 2).

Systolic blood pressure was lower (mean difference −0.6 mmHg, 95% CI −7.423 to 6.301) in the intervention group (137.8 mmHg) compared with the usual care group (138.4 mmHg). Similarly, hemoglobin A_1c_ was lower (a mean difference of −4.53 mmol/l, 95% CI −13.099 to 4.710) in the intervention group (57.2 mmol/l) in comparison to the usual care group (61.7 mmol/l), but for both of these clinical outcomes, this study was not intended to be powered to detect significant between-group differences. No statistically significant differences were found in quality of life between groups (*t*_112_=0.524; *P*=.60; 2-tailed).

Subgroup analysis found no differences between the respondents and the nonrespondents of each of the above-mentioned outcomes.

**Table 2:** Difference between intervention and usual care group in medication adherence at 3 months follow-up.

Outcome^a^			Intervention group		Usual care group		Test statistic (*df*)		*P* value (2-tailed)		Mean difference		95% CI
**Medication adherence**													
	Days adherence	6.85 (0.47)		6.36 (1.59)		2.37 (112)		.02		0.49		0.08 to 0.87	
	Percentage adherence	96.64 (5.60)		91.89 (18.59)		1.7 (112)		.05		4.74		−0.29 to 9.52	
	Repeat prescription	0.99 (0.11)		0.92 (0.21)		2.27 (116)		.02		0.07		0.00 to 0.12	

^a^Figures per complete case analysis. Data are reported as means (SD) and are not adjusted for any baseline characteristics. The numbers present the difference between the intervention and the comparator group in outcomes at 3 months follow-up. Positive numbers present outcomes that were larger among the intervention group than the comparator group, and negative numbers present outcomes that were smaller among the intervention group than the comparator group.

### Intervention Mechanism of Action

Multivariable regression analysis found that between-group differences in days of medication adherence was explained by changes in intentional and nonintentional nonadherence for the intervention group (beta.=074, SE=0.464; *P*=.04), but not for the control group (beta=.00, SE=1.35; *P*=.37); suggesting that changes in intentional and nonintentional nonadherence explained the improvements in medication adherence for the intervention group.

Further multivariable regression analysis revealed that better control of clinical outcomes (blood pressure <140/90mmHg or hemoglobin A_1c_ <42mmol/mol) was predicted by more days of medication adherence and positive beliefs about taking medication for the intervention group (beta=3.57, *P*=.03), but not for the control group (beta=.795, *P*=.46).

### Intervention Fidelity, Engagement, and Satisfaction

Intervention fidelity was 100% for the total duration of the intervention, suggesting that all messages were delivered as scheduled. Most (56/77, 73%) intervention group patients selected to receive 1 intervention message per day and some (21/77, 27%) selected to receive 2 messages per day. The majority of patients selected to receive intervention messages around the time they used to take their medications.

Total intervention engagement was 12 days, suggesting that patients interacted with the digital intervention and potentially engaged with the medication adherence intervention.

The majority of the participants found the intervention easy to use (51/70, 73%), liked the automated voice delivering the voice messages (44/70, 62.5%), the content of the messages (50/70, 71.5%), and the availability to call and ask questions when needed (36/70, 51.6%). Overall, patients were satisfied with the experience with the intervention (53/70, 76%), and they would recommend it to other people who take medications for a long-term health condition (46/70, 65%; Multimedia Appendix 2).

## Discussion

### Principal Findings

Among those patients with either or both high blood pressure and high glucose levels recruited from primary care practices, patients who used the digital intervention improved their adherence to medication at 3 months by an average of 2 days (intervention effect size Cohen d=0.42), compared with those continuing with their usual care only. Taking into consideration that adherence declines during the first month following usual care consultations [20], our trial suggests that the intervention can support treatment adherence to nonadherent patient.

The attrition rate was low, and the end of intervention clinical outcomes found that the intervention group patients had lower blood pressure and lower glucose levels, compared with those allocated in the control group. Although the study was not powered to assess effectiveness in clinical outcomes, considering the impact of medication nonadherence on uncontrolled blood pressure [21] and uncontrolled hemoglobin A_1c_ [22,23], these results provide us with confidence that the intervention is feasible and could potentially be an effective adjunct to primary care consultation.

The intervention effectively improved medication adherence by modifying patients' intentional and nonintentional nonadherence, which proves the importance of the highly tailored intervention to support health behavior change. This result proves that this highly tailored intervention can effectively support the processes of behavioral change and in turn the health behavioral outcomes.

It was also found that patients interacted with the digital intervention for 12 days, which provides us with confidence that patients engaged with the medication adherence intervention [24], a prerequisite to achieve behavior change and clinically meaningful outcomes at least in the short term.

### Strengths and Limitations

This trial provides evidence about the efficacy of the intervention to improve medication adherence based on self-reports, which might over estimate adherence levels. Nevertheless, the proxy measure of adherence, that is, the repeat prescription claims, was statistically significant, which provides us with confidence about the efficacy of the intervention; although both these measures do not directly reflect medication-taking behavior.

Another strength of the study is the recruitment of patients from a range of primary care practices, most in highly deprived areas. Considering the challenges with recruitment in a primary care setting [25], the recruitment rate provided us with confidence that the interventions can be scaled up to a wider range of practices and reach patients of different socioeconomic backgrounds.

A limitation of this study is that we could not reliably identify the patterns of nonadherent behavior (eg, frequency and sequence of nonadherent behavior) when patients were enrolled in the study. Similarly, we could not reliably identify the frequency of changes in prescribed medications, as well as the reasons for these changes (eg, therapeutic thresholds reported by patients or clinicians) [26]. Nevertheless, we decided to enroll all patients, regardless of the patterns or reasons of past nonadherence, to obtain more information about the intervention engagement and mechanism of action.

### Possible Mechanisms and Implications for Clinicians or Policymakers

The results of this study suggest that the intervention was effective at improving medication adherence by supporting intentional and nonintentional nonadherence, had good engagement and satisfaction, and low attrition rate. In view of the demographics of the participants, who had more experience with and possibly expectation of patient-clinician interactions, and the recruitment setting of the study [25], these findings prove that the digital intervention is a feasible and acceptable adjunct to primary care consultation.

The baseline face-to-face consultation lasted on average 30 min and involved the completion of study procedures and signposting patients to the digital intervention, in addition to usual care (eg, addressing patients' concerns and side effects). From those patients allocated to the digital intervention, only 1% requested additional human support regarding the use of this technology, and requests involved changes to the time of the message delivery, or the dose or the name of the medication when a change in the prescribed tablet was recommended by their health care providers. Moreover, the low attrition rate suggests that the intervention is highly acceptable as an adjunct to usual care. Given that the cost to deliver the digital intervention is very little (monthly cost of £0.029 per patient), and there were no adverse events or additional consultations recorded during the 3 months, it is likely that the intervention is inexpensive for the primary care.

### Future Directions

Improved medication adherence has been associated with decrease in all-cause mortality and morbidity, and digital interventions can be a solution to rapidly evolve the provision of health care [10]. Future research of larger sample sizes and objective outcome measures for medication adherence (eg, urine analysis for detection of antihypertensive medication) is needed to provide evidence about the effectiveness and cost-effectiveness of the intervention to support treatment adherence and clinical outcomes.

### Conclusions

This is the first trial that has been conducted in the primary care setting in the United Kingdom. The finding of this feasibility trial suggests that the intervention is a feasible adjunct to primary care consultations and effective in improving medication adherence and process of care to patients nonadherent to either or both antihypertensive and antiglycaemic medications. The intervention is scalable and low cost. More research is needed to evaluate its effectiveness and cost effectiveness.

## Appendix

Multimedia Appendix 1 Trial videos.

Multimedia Appendix 2 Usability, satisfaction and mechanisms of action.

Multimedia Appendix 3 CONSORT-eHEALTH (V 1.6.1).

## Abbreviations

NIHR: National Institute for Health Research
