# Supplementary material for: A Highly Tailored Text and Voice Messaging Intervention to Improve Medication Adherence in Patients With Either or Both Hypertension and Type 2 Diabetes in a UK Primary Care Setting: Feasibility Randomized Controlled Trial of Clinical Effectiveness
Source: J Med Internet Res. 2020 May 19;22(5):e16629. doi: 10.2196/16629 (PMC7267991; doi:10.2196/16629)
Supplement: Multimedia Appendix 1 [file jmir_v22i5e16629_app1.docx]

<https://vimeopro.com/healthandcarevideos/cambmed-maps>
