# Supplementary material for: A Highly Tailored Text and Voice Messaging Intervention to Improve Medication Adherence in Patients With Either or Both Hypertension and Type 2 Diabetes in a UK Primary Care Setting: Feasibility Randomized Controlled Trial of Clinical Effectiveness
Source: J Med Internet Res. 2020 May 19;22(5):e16629. doi: 10.2196/16629 (PMC7267991; doi:10.2196/16629)
Supplement: Multimedia Appendix 2 [file jmir_v22i5e16629_app2.docx]

| **Table 3**. Usability, satisfaction and mechanisms of action. | | | |
| --- | --- | --- | --- |
|  | disagree | neither  agree nor disagree | agree |
| **Usability, n (%)** | | | |
| The intervention was easy to use | 6 (8.5) | 13 (18.5) | 51 (73) |
| It would be useful, if the intervention was part of the usual care | 20 (28.7) | 26 (37.1) | 24 (34.2) |
| I would recommend the intervention to other people who take medications for long term health conditions | 9 (13.6) | 15 (21.2) | 46 (65.2) |
| **Satisfaction, n (%)** | | | |
| I am satisfied with the overall experience of the intervention | 5 (7) | 12 (17) | 53 (76) |
| I am satisfied with the voice delivering the voice messages | 11 (16) | 15 (21.5) | 44 (62.5) |
| I am satisfied with being able to contact the intervention | 4 (6) | 30 (42.4) | 36 (51.6) |
| I am satisfied with the content of the intervention messages | 6 (8.5) | 14 (20) | 50 (71.5) |
| I am satisfied with being able to ask very personal things using the intervention (inbound calls) | 1 (1.5) | 34 (48.5) | 35 (50) |
| I am satisfied with being able to ask about things that are troubling me | 3 (4.3) | 41 (59) | 26 (36.7) |
| I am satisfied with the responses I received from the intervention (interactive messages) | 2 (3) | 36 (51.5) | 32 (45.5) |
| I am satisfied with the responses I received to my personal queries/questions | 2 (3) | 34 (48.5) | 34 (48.5) |
| I am satisfied with the cost of the intervention | 2 (3) | 38 (54.5) | 30 (42.5) |
| **Mechanisms of action, n (%)** | | | |
| The intervention messages addressed my personal needs | 15 (21.5) | 25 (36) | 30 (42.5) |
| The intervention messages helped me to take all my tablets as prescribed | 15 (21.5) | 16 (22.9) | 39 (55.6) |
| The intervention messages reminded me to take all my medication as prescribed | 17 (24.4) | 14 (20) | 39 (55.6) |
| *Data are presented in number of participants and percentages.*  *Responses from n=70 intervention group patient at follow up questionnaires.* | | | |
